# USA300 *Staphylococcus aureus* persists on multiple body sites following an infection

**DOI:** 10.1186/s12866-018-1336-z

**Published:** 2018-12-05

**Authors:** Timothy D. Read, Robert A. Petit, Zachary Yin, Tuyaa Montgomery, Moira C. McNulty, Michael Z. David

**Affiliations:** 1 Department of Medicine, Division of Infectious Diseases, Emory University School of Medicine, Atlanta, GA USA; 2 Department of Human Genetics, Emory University School of Medicine, Atlanta, GA USA; 3 Department of Pediatrics, Section of Infectious Diseases, University of Chicago, Chicago, IL USA; 4 Department of Medicine, Section of Infectious Diseases and Global Health, University of Chicago, Chicago, IL USA; 5 Department of Medicine, Division of Infectious Diseases, University of Pennsylvania, Philadelphia, PA USA

**Keywords:** MRSA, SSTI, USA300, Colonization, Plasmids, Phage, Antibiotic resistance, *Staphylococcus aureus*

## Abstract

**Background:**

USA300 methicillin-resistant *Staphylococcus aureus* (MRSA) is a community- and hospital-acquired pathogen that frequently causes infections but also can survive on the human body asymptomatically as a part of the normal microbiota. We devised a comparative genomic strategy to track colonizing USA300 at different body sites after an initial infection. We sampled ST8 *S. aureus* from subjects at the site of a first known MRSA infection. Within 60 days of this infection and again 12 months later, each subject was tested for asymptomatic colonization in the nose, throat and perirectal region. 93 *S. aureus* strains underwent whole genome shotgun sequencing.

**Results:**

Among 28 subjects at the initial sampling time, we isolated *S. aureus* from the nose, throat and perirectal sites from 15, 11 and 15 of them, respectively. Twelve months later we isolated *S. aureus* from 9 subjects, with 6, 3 and 3 strains from the nose, throat and perirectal area, respectively. Genome sequencing revealed that 23 patients (ages 0–66 years) carried USA300 intra-subject lineages (ISLs), defined as having an index infection isolate and closely related colonizing strains. Pairwise distance between strains in different ISLs was 48 to 162 single nucleotide polymorphisms (SNPs) across the core regions of the chromosome, whereas within the same ISL it was 0 to 26 SNPs. Strains in ISLs from the same subject differed in plasmid and prophage content, and contained deletions that removed the *mecA-*containing SCC*mec* and ACME regions. Five strains contained frameshift mutations in *agr* toxin-regulating genes. Persistence of an ISL was not associated with clinical or demographic subject characteristics. We inferred that colonization with the ISL occurred about 18 weeks before the first assessment of asymptomatic colonization.

**Conclusions:**

Clonal lineages of USA300 may continue to colonize people at one or more anatomic sites up to a year after an initial infection and experience loss of the SCC*mec*, loss and gain of other mobile genetic elements, and mutations in the *agr* operon.

**Electronic supplementary material:**

The online version of this article (10.1186/s12866-018-1336-z) contains supplementary material, which is available to authorized users.

## Background

*Staphylococcus aureus* is one of the most common human bacterial pathogens, causing skin and soft tissue infections (SSTI), bacteremia, osteomyelitis and other disseminated infections [1]. While it can be deadly, *S. aureus* is more commonly a commensal species, living on the skin, the mucous membranes, and in the gut [2] of 25–50% of the human population [3]. Since the 1940s, the species has evolved resistance to each class of antibiotics introduced to treat infections [4]. Resistance to β-lactam antibiotics due to the expression of altered penicillin-binding protein PBP2a, encoded by *mecA* and *mecC* genes located in a mobile genetic element called SCC*mec* [5, 6], has been a particularly vexing problem. Strains that contain PBP2a, known as methicillin-resistant *S. aureus* (MRSA), have proliferated since being first reported in 1961 in the United Kingdom [7, 8].

Previous studies have demonstrated differences in fitness and virulence between genetic backgrounds of MRSA [9–11]. One genotype of MRSA, USA300, emerged in the United States (US) in the 1990s and spread rapidly in the community to become the leading cause of SSTIs in US urban emergency departments by 2004 [12]. USA300, which has also spread to many other parts of the world [13, 14], is MLST ST8 and usually carries SCC*mec* type IV, the phage-encoded Panton-Valentine leukocidin (PVL), and the arginine catabolic mobile element (ACME) [15, 16]. The genetic factors underlying the meteoric success of this strain type are not completely understood but it is likely that enhanced virulence promoting transmission, tolerance of the human body to its presence as a commensal, and an apparently limited fitness cost of its antibiotic resistance [17] are important. Only a few studies have suggested that USA300 can stably colonize patients for more than 6 months, but these have been limited by using genotyping methods that lack the genetic resolution to distinguish between persistent colonization by a strain and acquisition of a new colonizing strain from a different lineages within the same sequence type [18]. However, two recent studies have used whole genome sequencing to demonstrate the persistence of single USA300 clones to cause recurrent infections in individuals [19, 20].

There have now been a number of comparative genomics studies on USA300 MRSA. These have included studies using the fine scale resolving power of genomics to characterize the global population structure [13, 14, 21–23] and transmission patterns between patients [24, 25], within households [26–28] and in recurrent SSTIs [19]. In this work we explored the genetic diversity of USA300 on the bodies of subjects during a year of surveillance after an initial, clinically significant USA300 MRSA infection. In individual subjects within a prospective cohort, we set out to determine whether a single USA300 clonal lineage persisted after an infection, whether it persisted on or spread among three body sites, and how the genome changed during the year of observation.

## Methods

### Strain isolation and phenotypic testing

169 adult and pediatric (< 16 years of age) patients with a first known MRSA infection at any anatomic site were enrolled at the University of Chicago Medical Center (UCMC) in a longitudinal study of risk factors for recurrent MRSA infections between April 2012 and February 2014. The MRSA isolate from the site of infection is hereafter called the “initial infection isolate”. Subjects underwent an enrollment visit within 60 days of culture of their initial infection (time 1) when they were tested for *S. aureus* colonization of the nares, throat, and perirectal region. Subjects returned approximately one year later and were again cultured at the same 3 sites (time 2). Overnight broth enrichment culture (tryptic soy broth with 7% NaCl at 37 °C) was used to isolate *S. aureus* from colonization culture swabs. The broth was then plated on selective colorimetric solid media (Chromagar *S. aureus*) to initially screen for the presence of *S. aureus*, and the species was confirmed by the presence of the signature *spa* gene by PCR[29]. All *S. aureus* isolates, including the initial infection isolate and up to a single isolate obtained from each colonization culture, were frozen at − 80 °C. All of these isolates (up to 7 from each subject) underwent genotyping by MLST (expecting ST8 for USA300), PCR for the presence of the PVL genes, and, for MRSA isolates, SCC*mec* typing (expecting type IV for USA300). These are standard markers for identification of most USA300 MRSA strains [16]. All strains produced hemolytic clear zones on blood agar plates. Initial infection isolates were phenotypically tested for susceptibility to clindamycin, erythromycin, gentamicin, linezolid, methicillin, rifampin, tetracycline, trimethoprim-sulfamethoxazole and vancomycin. A medical record review was performed to determine the site of the initial MRSA infection and to determine if a true infection was present [30], the therapy used to treat infection, demographics, and comorbidities. Each subject was administered a detailed survey to assess risk factors for MRSA infection (Additional file 1).

All 31 subjects among the 169 enrolled subjects who had an ST8 MRSA initial infection isolate and one or more ST8 *S. aureus* isolates obtained from colonization cultures at one or both colonization culture visits were studied (*n* = 31/169, 18.3%). Three of the initial infection isolates were lost, leaving 28 of these isolates from 31 patients. 93 isolates (initial infection and colonization) from these 31 subjects underwent genomic preps and Illumina sequencing.

### Illumina sequencing

Genomic preps were prepared with the DNeasy Blood and Tissue Kit (Qiagen, Germantown, MD). Libraries for sequencing 93 strains were constructed using Nextera technology (Illumina, Inc., San Diego, CA). Libraries were sequenced on an Illumina HiSeq instrument using either 100 nt or 125 nt paired-end protocols.

### Sequence data analysis

Two strains had less than 100× predicted genome coverage (assuming an average 2.9 Mbp chromosome sequence). For consistency, all other genomes were downsampled to 100× coverage before assembly using a custom script (https://gist.github.com/rpetit3/9c623454758c9885bf81d269e3453b76) based on the seqtk toolkit (https://github.com/lh3/seqtk). Three strains were found not to be ST8 by genome-based MLST and were not included in subsequent analysis. Strains were assembled de novo using SPAdes [31] and annotated using Prokka [32]. Antibiotic resistance phenotypes were predicted for each strain based on the methods of Gordon et al. [33]. SnpEff 4.2 [34] was used to annotate SNPs in the VCF output of the parsnp alignment based on USA300_TCH1516 (NCBI RefSeq accession NC_010079.1; isolated from a pediatric patient at the Texas Children’s Hospital before 2007 [35]) coordinates. SNPs were mapped back on the shortened alignment using R BioStrings [36]. USA300_TCH1516 proteins were mapped to their orthologs in the *S. aureus* type strain NCTC8325 [37] using blastp [38], and gene ontology enrichment was performed using PANTHER [39] in December 2016.

A whole genome alignment of all the newly sequenced USA300 genomes was performed using Parsnp v1.2 [40] against the closed reference genome USA300_TCH1516. The Snippy pipeline [https://github.com/tseemann/snippy] was used to cross-validate SNPs predicted by Parsnp and to identify frameshifts and small indels. Plasmid content was found to be variable between strains (discussed below), and therefore not useful for comparisons across the whole dataset. One strain (an initial infection isolate) fell outside of the USA300 subgroup and was therefore excluded from the rest of the study, leaving a total of 89 strains. Due to these exclusions (see Additional files for a summary diagram), 2 patients were not represented by any samples, and we therefore concentrated our analysis on the 29 remaining patients, 28 of whom had an USA300 initial infection isolate. From the Parsnp alignment of the 89 strains, we extracted a core region of the USA300 chromosome of 2471 kbp (86%). The 14% of the chromosome excluded because it was missing in at least one of the genomes included SCC*mec*, ACME, the SaP15 pathogenicity islands, and the SLT and βC prophages [35]. Other regions not part of the core alignment were plasmids, rRNA operons, transposons, portions of membrane proteins and non-genic repeats.

The concatenated common aligned portion (2471,416 of the 2,872,915 bp [86%] USA300_TCH1516 reference [Additional file 2]) was used as the input for the recombination detection program ClonalFrameML v1.0–19-g9488a80 [41], along with a maximum likelihood guide tree produced by RAxML [42] v7.2.8. We removed 10,884 bp from the alignment identified by ClonalFrameML [41] as possibly of recombinant origin and then used as input for a maximum likelihood phylogeny under the GTRGAMMA model with 100 bootstraps using RAxML. Noisy (v1.5.12) [43] was used to scan for potentially homoplasious sites in the alignment. The sequences of the inner nodes of the phylogeny were reconstructed using FastML [44], based on the post-ClonalFrameML trimmed alignment. The R ape [45] dna.dist function was used to find the number of SNPs between leaves and ancestral nodes of the tree based on Hamming distance.

To identify integrated prophages, we aligned PCR primer sequences from seven diverse classes of *S. aureus Siphovirus* integrase [46] using blastn (version 2.3.0)[38], with the ‘blastn-short’ task option. To identify plasmid contigs, we aligned against a database of 230 complete *S. aureus* plasmids downloaded from NCBI using the blastn ‘megablast’ option. The contigs were also aligned against sequences from a Gram positive plasmid replicon typing scheme [47], identifying plasmid replicon [47] matches with blastn (task = blastn-short, cutoff = 1 mismatch or gap, match of 19 bases or more).

Raw sequence data were deposited at NCBI SRA under BioProject accession PRJNA388087.

### Association of Subject Characteristics with persistence of an intra-subject lineage (ISL) of *S. aureus*

We defined an ISL as a cluster of isolates isolated from a patient that derived from a recent last common ancestor suggestive of a transmission bottleneck. Based on analysis of the data (described in the Results), members of an ISL isolated all shared < 30 SNPs in their core genome regions. We performed univariate testing (chi square, Fisher exact, Wilcoxon rank-sum or t-tests) to determine if subject characteristics (see list in  Additional file 1) were associated with colonization with an ISL (see discussion of ISL below) after initial infection at time 1 and time 2. We used Bonferroni correction to compensate for multiple comparisons.

## Results

### Colonizing ST8 *S. aureus* strains can be isolated 12 months following initial infection

We isolated and sequenced 89 novel USA300 strains from 29 subjects who presented with a ST8 MRSA infection and were subsequently found to have at least a single ST8 *S. aureus* isolate obtained from a colonization culture at ≥1 of the 3 tested body sites at time 1 (enrollment) or time 2 (12 months) (Additional file 3). One strain, derived from a single, randomly chosen colony on the culture plate, was selected to represent each time and body site. Study subjects received 0–6 (median 1, interquartile range [IQR], 1–2) antibiotic drugs during treatment for the index infection (Additional file 1). *S. aureus* initial infection isolates (i.e., cultured from the site of infection) were retained prospectively for further study and underwent whole genome sequencing. Nose, throat and perirectal body sites were cultured at time 1, and *S. aureus* was isolated from 16, 15 and 17 patients, respectively. Approximately 12 months after the initial visit (time 2), we isolated *S. aureus* from 6, 3 and 4 subjects from nose, throat and perirectal sites, respectively (Table 1 and Additional files 1 and 3). While all initial infection isolates were obtained from suspected sites of infection, on review of the medical record, 2 were found to be obtained from sites of colonization and did not require treatment.

**Table 1:** Demographic and clinical information, indicating for each subject the presence (y) or absence (n) of an intra-subject lineage (ISL) and its detection (y) or absence (n) at time 1 (enrollment) and time 2 (1 year follow-up) among colonizing *S. aureus* isolates. Additional demographic and clinical variables are presented in the Supplemental data

Patient ID	Initial Infection Isolate	ISL	Time 1^a^	Time 2^a^	Sex	Age range (years)^b^	Initial Infection
1	y	y	y	y	female	51–55	respiratory
2	y	y	y	n	female	0–5	perirectal abscess
3	y	y	n	y	female	0–5	neck abscess
4	y	y	n	y	male	0–5	right buttock abscess
5	y	y	y	n	female	11–15	draining skin abscess
6	y	y	y	n	female	56–60	abdominal wall abscess
7	y	y	y	n	male	6–10	left groin abscess
8	y	y	n	y	male	0–5	pleural fluid culture
9	y	y	y	y	male	46–50	right ear drainage
10	y	n	y	y	female	31–35	abscess right shoulder
11	y	y	y	y	male	6–10	left ear drainage
12	y	y	y	n	male	0–5	abscess near anus
13	y	y	y	y	female	0–5	left labial cellulitis and abscess
14	y	y	y	n	female	0–5	left labial abscess
15	y	y	y	n	female	41–45	drainage from psoriatic plaque
16	y	y	y	n	female	51–55	foot wound
17	y	n	y	n	male	0–5	left inguinal abscess
18	y	y	y	y	female	56–60	sputum culture
19	y	y	y	y	female	66–70	left leg wound drainage
20	y	y	n	y	female	0–5	wound drainage neck, axilla, groin
21	y	n	n	n	male	0–5	buttock abscess
22	y	y	y	n	male	0–5	culture dorsum right hand
23	y	n	n	n	female	41–45	right facial abscess
24	y	y	y	n	female	31–35	left upper arm abscess
25	y	y	y	n	male	6–10	wound drainage, scrotal abscess
26	y	n	n	n	male	36–40	unknown
27	y	y	y	n	female	36–40	wound drainage leg abscess
28	y	y	y	n	male	26–30	wound drainage chin abscess
29	n	n	y	n	male	0–5	respiratory

^a^indicates whether or not a USA300 strain within an ISL was isolated at time 1 (enrollment) or time 2 (1 year later); “y” indicates the presence of the ISL at the indicated visit, and “n” indicates that the ISL was not isolated at the indicated visit

^b^age at enrollment (time 1) visit

### Phylogeny of USA300 isolates from Chicago patients reflect history of the local epidemic

**Fig. 1:**
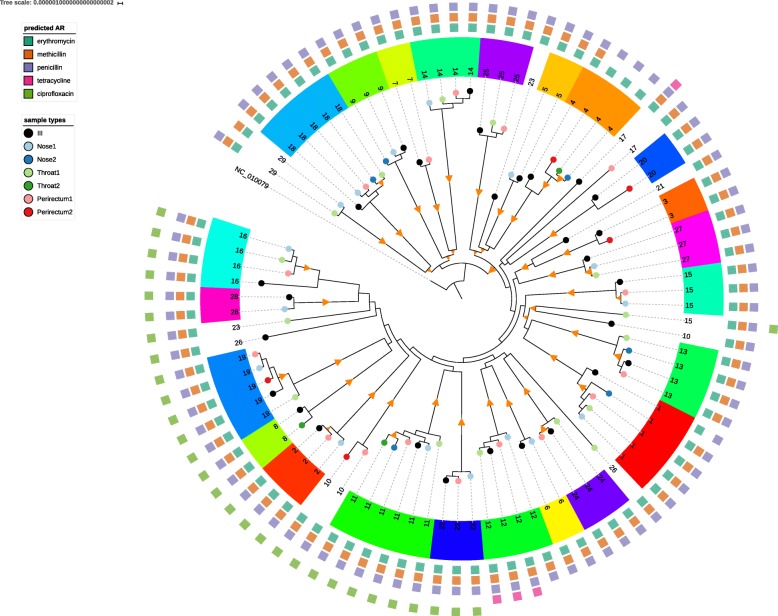
Maximum Likelihood tree of USA300 strains sequenced in the project. Orange triangles are branches with > 80–100% bootstrap support. Tips of trees are labelled by site of isolation (black = index infection isolate (III), green = throat, blue = nose, red = perirectal). Light colors are from sampling time 1, dark colors from sampling time 2. A color code and patient number were assigned to each patient. The squares on the outer ring represent antimicrobial class resistance predicted by the genome sequence. The figure was drawn using iTOL software [74]

The phylogeny of the core region of the 89 USA300 strains (Fig. 1) was composed of clades of leaf nodes with short branches near the tips, mostly representing isolates from individual patients, separated by relatively long branches in the middle section. In the deepest parts of the tree, branches were shorter and had lower bootstrap confidence. This pattern, seen in other genome-based studies of USA300 [26, 27] reflected the history of the epidemic [48]. USA300 spread rapidly after its introduction in the 1990s and achieved a large population size. USA300 then persisted in the community, causing sporadic clinically significant infections. By rooting the tree with the TCH1516 reference, a group of strains with mutations in *gyrA* and *grlA* predicted to confer ciprofloxacin resistance fell into a late-branching subgroup. The later development of resistance to fluoroquinolones was in line with results from analysis of the global population of USA300 [13, 22, 26, 27].

### USA300 strains form “intra-subject lineages” (ISLs) present on multiple anatomic sites of a Subject’s body

As mentioned above, in most cases, the strains isolated from one subject clustered into a monophyletic group with shorter branch lengths than strains from other patients. We termed these groups ISLs, and we inferred that all strains within them derived from a single recent transmission bottleneck. The initial infection isolate from one subject (29) was lost during the course of the study, and we treated the remaining strains as outside of an ISL. Based on the phylogeny we removed six strains from ISLs because they were obviously phylogenetically unrelated to the other strains in the ISL. Five subjects (10,15,17,23,26) had colonizing strains from a different ISL than the initial infection isolate, suggesting colonization with more than one USA300 lineage. After removing these non-conforming strains, we were left with 23 subjects with an ISL that included an initial infection isolate and at least one other body site isolate*.*

Of the 23 patients with a USA300 ISL, 15, 11, and 15 (54, 39 and 50%) at the time 1 (enrollment) visit had an an isolate from the same ISL in the nose, throat and perirectal area, respectively. Following up one year later (i.e., time 2), 6 (21%) patients still had a strain from the same ISL in the nose, and 3 (11%) each in the throat in the perirectal area (Additional file 3). Strains were isolated at time 2 in 9 of the of original 23 ISLs (Table 1). Fourteen subjects had > 1 body site colonized by a strain from within an ISL at enrollment (time 1); only 2 subjects were colonized at > 1 body site with an ISL a year later.

In univariate analyses, after Bonferroni correction, we found that no tested subject characteristics, including age, sex, type of insurance coverage, race, treatment of the index infection with clindamycin, with vancomycin or with trimethoprim-sulfamethoxazole, or pet exposure (see complete list in Additional file 1) were significantly associated with colonization with an ISL after index infection at time 1 or time 2.

### Genetic distance between and within ISLs

**Fig. 2:**
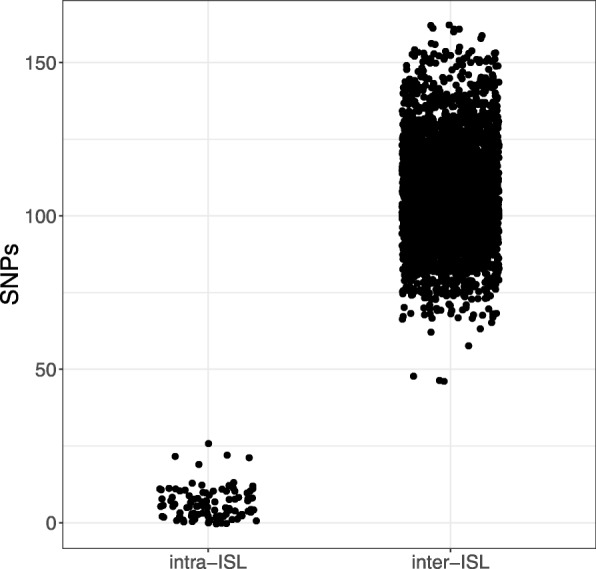
Distribution of the number of SNPs separating strains within the same intra-subject lineage (ISL) compared with other strains

For each strain we calculated the number of SNPs between every other strain in another ISL and compared it to the number of SNPs within an ISL (Fig. 2). Strains in different patients had from 48 to 162 SNP differences (median = 106). The range for strains in the same ISL was 0 to 26 (median = 5).

**Fig. 3:**
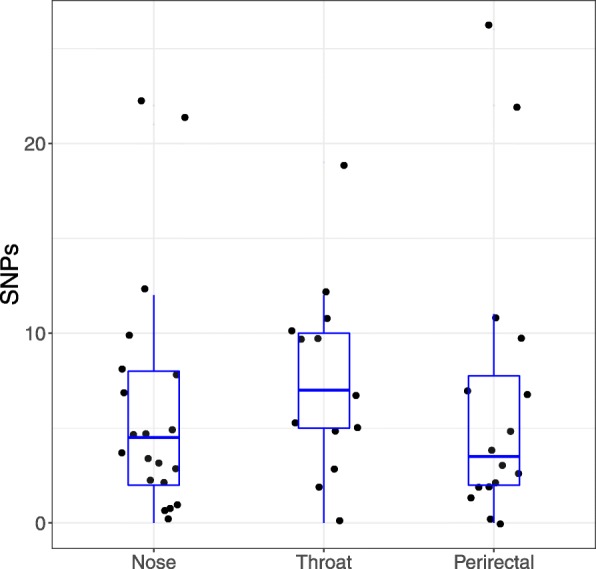
Number of SNPs separating the initial infection isolate and strains within the same intra-subject lineage (ISL) at different sites. Horizontal lines in the box show the median, and box hinges are the 25th and 75th quartile. The upper whisker extends from the hinge to the largest value no further than 1.5 * interquartile range (IQR) from the hinge. The lower whisker extends from the hinge to the smallest value at most 1.5 * IQR of the hinge

The intra-ISL relationships were further analyzed by measuring the number of SNPs between each strain and the initial infection isolate (Fig. 3). There was no significant pattern to the number of SNPs differing in the initial infection isolate and each of the 3 tested body sites (ANOVA *p* = 0.8). The ranges for each body site were: nose, 0–22 (median 4.5), throat, 0–19 (median 7) and perirectal 0–26 (median 3.5). As expected, within host analysis showed that there were a significantly greater number of SNPs within ISLs between the initial infection isolate and strains from time 2 (range 2–26: median 10) compared with time 1 (range 0–12: median 3) (t-test, *P* < 7.8e-7).

**Fig. 4:**
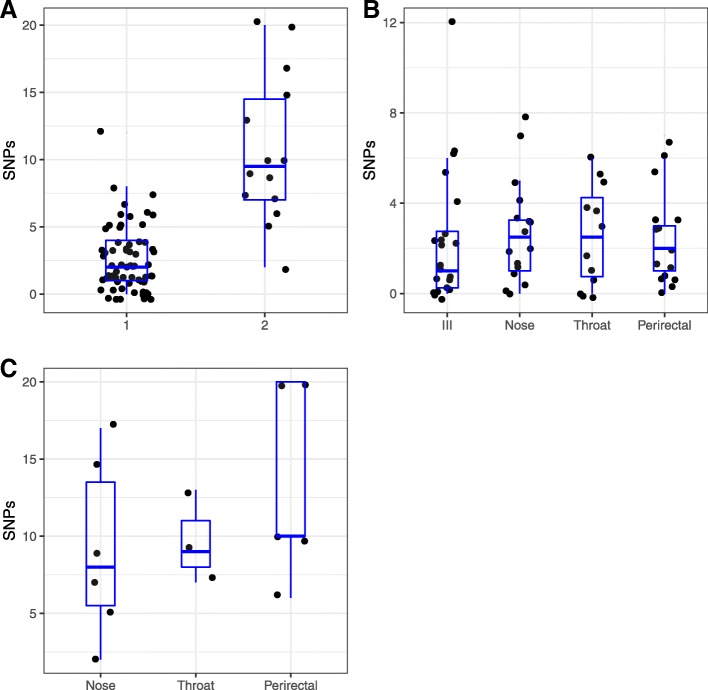
Number of SNPs between the most recent common ancestor (MRCA) and strains at each sampling time point: **a** aggregated for each time point. Sampling time point 2 was approximately 1 year after time point 1. **b** at time point 1 between the MRCA and each body site. **c** at time point 2 between the MRCA and each body site (note difference in y-axis scale between B and A/C)

We used FastML [44] to reconstruct the sequence of internal nodes of the phylogeny and then found the number of SNPs differing between each strain of an ISL and its most recent common ancestor (MRCA). There was a significantly greater number of SNPs from the MRCA at time 2 (median = 9.5; interquartile range [IQR] = 7–14.5) compared with time 1 (median = 2; IQR = 1–4) (t-test, *p* < 7.7 e-13) (Fig. 4a). There was no significant difference in the number of SNPs between the initial infection isolate or other body sites at time 1 (ANOVA, *p* = 0.95), or at the 3 body sites sampled at time 2 (ANOVA, *p* = 0.49) (Fig. 4b and c).

### Shifts in Mobile genetic element composition within ISLs

In *S. aureus* changes in accessory gene content mediated by prophage, genome island insertion and plasmid transfer are important mechanisms for genome evolution. However, tracking these changes is problematic using short read shotgun data. Loss or gain of a single element might affect dozens of genes at once, and plasmids and prophages undergo frequent recombination and share common sequences, making identification difficult [49]. Nevertheless we were able to use previously developed typing schemes to gain insights into changes in mobile element composition during colonization of the studied subjects.

For prophages, we used the scheme of Goerke et al., who defined 7 distinct sequence types of *S. aureus* integrase (Sa1–7) [46]. Presence of the each class was called based on BLAST matches of at least one of the integrase-specific primers. Most strains had Sa2 (87/89; 98%) and Sa3 (86/89; 96%) family prophages. These correspond to the ɸSa2 (ɸSLT) and ɸSa3 (ɸBC) prophages in the USA300_TCH1516 reference genome, respectively [35]. In addition, 40/89 (45%) strains had a Sa5 integrase gene. Eight strains (9%), all from patients 13 and 15, carried an Sa1 integrase. The patterns of prophage carriage mirror those described in USA300 by Jamrozy et al. [22], with the exception that we did not identify the rarer ɸSa7 prophages in our samples. We noted four occurrences of prophage composition change within an ISL (Additional file 3). These were the apparent loss of ɸSa3 in the initial infection isolate of subject 18 and the first throat culture of subject 15 and the apparent gain of ɸSa5 in the first perirectal isolate of subject 10 and the initial infection isolate of subject 16 (although based on its deep branching, the initial infection isolate may represent a separate colonization event from other strains isolated from subject 16).

To examine plasmid content we used the combination of BLAST alignments against DNA sequences from the Gram positive replicon typing scheme of Lozano et al. [47] and 344 complete *Staphylococcus spp.* plasmid sequences downloaded from NCBI (Additional file 3). All genomes contained the common backbone region of the 27 kb antibiotic-resistance plasmid of TCH1516, pUSA300HOUMR (NC_010063) [35]. 67/89 (72%) strains matched against > 25 kb of the plasmid, the rest contained subsequences of the plasmid as small as 13 kb that all contained the pN315-like replicon [50]. Eight strains contained the backbone of the 37 kb pUSA03 [51] multidrug resistant conjugative plasmid sequence but with the *mupA* gene missing (*mupA* encodes an altered isoleucyl-tRNA synthetase that confers mupirocin resistance). In the Jamrozy et al. study, in contrast to our findings, 146/154 (95%) USA300 strains from SSTIs carried the pUSA300HOUMR backbone plasmids and 5% (8/154) carried pUSA03-like plasmids. Families of smaller (< 10 kb) plasmids were present in some of our strains. The cryptic pUSA01 plasmid was found in 57 (64%) strains; pC194-like plasmids [52] were found in 6; 4 strains carried a plasmid with tetracycline-resistance gene *tetK* similar to the pUSA02 plasmid [51] (NC_007791); and there were 3 and 1 strains with plasmid replicons of the pUB110 [53] and pWG745 [54] families, respectively. Nine ISLs had strains with different plasmid content (patients 1, 2,6,7,8,9,11,12,16; Fig. 1). Plasmid content was not linked to a particular body site or sampling time point.

### Predicted antibiotic resistance variation within ISLs

We observed that strains within several ISLs varied in their antibiotic resistance profile, based on the genomic predictions using the method of Gordon et al. [33]. Phenotypic susceptibility data for the index infection isolates is shown in Additional file 4. The first throat isolate of subject 15 was predicted to be ciprofloxacin resistant through *gyrA/grlA* mutations. This isolate was not part of the same ISL as the initial infection, nose and perirectal isolates from subject 15 (Fig. 1), and therefore it was not possible to determine whether the mutations occurred during colonization of subject 15 or earlier. Subject 15 was an outpatient who received only clindamycin during treatment of the index infection.

Two subjects had strains containing a 4.5 kb plasmid > 99% similar to pT181 [55] (NC_001371.1) and pUSA02 [51] (NC_007791), with the *tet*K tetracycline-resistance determinant. One of these was the initial infection isolate of subject 17, which was in a different ISL from the other strain isolated from this person (Fig. 1). From subject 12, the initial infection isolate and 2 other strains contained the *tet*K plasmid, but the nose strain did not. Either the strain originally infecting the subject was tetracycline-resistant and the nose isolate had been cured of the plasmid, or the plasmid was acquired by the ancestor of the three tetracycline-resistant strains. Both scenarios could be considered equally parsimonious as they require one plasmid gain/loss event. Neither subject 12 nor 17 were recorded as being treated with a tetracycline.

In subject 29, who was not treated with a β-lactam antibiotic for the index infection, the nose isolate had lost its *blaZ* gene owing to a deletion. The throat isolate from subject 29 was identical in its core chromosome sequence and had a plasmid with no deletion. In two subjects, strains switched from MRSA to MSSA because of deletion of the SCC*mec* cassette, which included the *mecA* gene. In subject 4 the index infection isolate was MRSA but all three strains isolated at time 2 (the 1-year visit) were MSSA. For subject 1, the situation was reversed. The index infection isolate was MSSA, but the colonizing strains isolated were all MRSA. The most parsimonious explanation was loss of the genes in the ancestor of the initial infection isolate shortly after colonization of the subject. Neither subject 1 nor 4 was treated with a β-lactam antibiotic for the index infection.

### SNP patterns do not show signatures of strong positive selection within ISLs

Of the 1882 SNPs in core regions, 1494 (79%) were in genes and of these, 1101 (73%) caused non-synonymous or premature termination changes. This high percentage of mutations affecting amino acid sequences is typical when comparing closely related bacteria before purifying selection has time to operate [56] and has been reported previously in studies comparing USA300 strains [19, 22]. The non-synonymous changes fell over 787 genes. Using a Poisson test, we did not find a significant deviation from the number of mutations that would be expected based on the gene length, if the mutations were occurring randomly across the genome. The gene with most changes was USA300HOU_1372, encoding an extracellular binding protein precursor (*ebp*) with 27 mutations; this protein is also the largest in the genome (10,421 amino acids). USA300HOU_0192 encoding a non-ribosomal peptide synthase (2397 aa), had the second most changes with 7 mutations. Most mutations were only found in one strain and more than 95% were found in 7 or fewer genomes. This was a result of the sampling strategy employed in the study, where a maximum of seven strains was taken from the same subject (one initial infection isolate plus up to one isolate colonizing each of 3 body sites at 2 time points). Mutations that occurred frequently were those found only in the reference (and hence common to all 89 strains sequenced here), in deeper branches of the tree, and those causing ciprofloxacin resistance.

We were interested to determine if there were any patterns in terms of the function of genes that acquired SNPs only in a certain body site, as it was possible that there was tissue-dependent selection that may act over multiple genes linked by common phenotype. We found 50, 190, 187 and 246 SNPs unique to nose, perirectal area, throat and initial infection isolate, respectively. Using PANTHER [39] we looked for pathways in the Gene Ontology enriched for genes affected by terminations and non-synonymous mutations at each site but found no significant results after multiple hypothesis correction.

### Deletions in Agr and ACME/SCC*mec*/ɸSa2 deletions within ISLs

We found that chromosomal indels reshaped virulence and resistance elements in the initial infection isolates and in the colonization strains. ACME is a 31 kb mobile genetic element that may be important for the success of North American USA300 strains [57] (although it absent from South American strains) [21]. Eleven strains in this study were found to have a deleted ACME and one partially deleted. In 6 subjects, the ACME element appears to have been lost by strains within the ISL (assuming the MRCA of the ISL was ACME+). In subject 25, who was treated with trimethoprim-sulfamethoxazole for the index infection, all strains were deleted in their ACME element and also lost their flanking SCC*mec* cassette, making them MSSA. Two strains isolated from the perirectal region of subject 20 were missing the ɸSa2 prophage carrying the PVL *lukSF* genes. These accessory genes are postulated to be important for SSTIs caused by USA300 (discussed in [58]).

We also noted that 5 strains contained frameshift mutations in the *agr* regulatory locus that would theoretically render them less capable of causing toxin-mediated tissue damage [59, 60] (Additional file 3). However, these strains and all others isolated for this study were hemolytic when grown on blood agar plates, suggesting that these mutations did not completely disrupt the system. Two strains from subject 26 (initial infection isolate and first throat isolate) that were not part of the same ISL had frameshift mutations in different portions of the *agrC* gene. Three of six strains from subject 11, on a monophyletic branch of the ISL phylogeny carried the same frameshift in the *agrA* gene encoding the regulatory protein, suggesting a single mutation event. This suggested that functional products of *agr* were not necessary for long-term survival on patients. Also, the mutant strains were isolated at both timepoints in all three body sites, thus demonstrating that *S. aureus* strains had migrated among anatomic sites on this subject.

## Discussion

In this study we showed that *S. aureus* USA300 ISLs, defined as having < 30 SNPs over 86% of the chromosome from the common ancestor, asymptomatically colonized a patient for more than a year after a confirmed or suspected infection. This cutoff is arbitrary and not meant to define an absolute limit but it is line with numbers of SNPs per year reported in other recent studies of *S. aureus* carriage. Young et al. [61] sequenced 68 nasal and blood isolates from a MSSA clone in one patient over 6 months, finding a total of 30 SNPs in all isolates. Sabat et al. reported that there were a maximum of 8 SNPs difference between USA300 isolated at the same time from different body sites of patients during a USA300 outbreak in Suriname [25]. Our finding here of temporal stability within a single USA300 lineage for a year or more also echoes the result of the shotgun metagenomic study of Oh et al. [62], who found that strain-specific SNPs for another human colonizing *Staphylococcus* species, *S. epidermidis*, could be recovered at the same skin sites at sampling times 1–2 years apart. Some subjects were colonized with more than one ISL: in 5 of the 29 subjects, we isolated USA300 from different lineages, and in 2 we isolated colonizing isolates that were not USA300 (one was another ST8, the other was ST6).

There have been few prior studies of longitudinal carriage of MRSA with whole genome sequencing to assess persistence of clonal carriage. To the best of our knowledge, there have been no previous such studies in which multiple body sites have been tested along with genome sequencing of an initial infecting isolate. In addition, comparison of *S. aureus* ISLs at different body sites over time is also novel in the literature. These data have therefore enabled us to perform a number of novel analyses on clonal variation within ISLs.

For example, we found that only a median of 2 SNPs separated the ISL isolates from the predicted MRCA sequence (Fig. 4a). The small number of SNPs suggested a short time period after the transmission bottleneck event, which may be a feature of the high virulence of USA300 [58]. Multiple genome-based studies have estimated the *S. aureus* mutation rate to be between 1.3–3.3 × 10^− 6^ SNPs per genome per year [26, 63–66], or approximately 1 SNP every 9 weeks [2]. Based on this estimate, the median time from MRCA to first time point (time 1) strains would have been a median of about 18 weeks with an IQR of 9–36 weeks. However, because of the limited isolate sampling, this timing is likely an underestimate. When the same patient was sampled a year later (time 2), the chance of culturing an ISL isolate was lower than time 1, which suggested that asymptomatic carriage was declining. Comparison of the positions on the ISL phylogeny of strains from the first and second time point for strain isolation indicated that it was possible that strains may have moved between or among body sites. These results also suggested that the nose, throat and perirectal area were approximately equally efficient at maintaining *S. aureus* colonization. Other studies have come to different conclusions, pointing to particular anatomic sites as potentially more important for long-term colonization than others, for example the anterior nares or the oropharynx [67, 68]. This finding challenges the traditional assumption that the anterior nares is the site of long-term carriage in colonized subjects. However, additional studies, sampling patients over longer time periods, are needed to understand the typical lifecycle of an ISL on a human subject. This information, in turn, may be important in the design of decolonization studies and regimens.

We did not identify a specific gene or locus that acquired an unusual number of SNPs suggestive of positive selection. With the number of genomes in our study, we had limited power to detect parallel mutations, especially those that may have been associated with adaptation specifically to the anatomic niches of the nose, throat or perirectal region. We did, however, encounter a significant number of genetic changes associated with movements of mobile elements during the history of colonization, including phage and plasmid gain and loss and loss of ACME and SCC*mec* elements. The latter two are particularly significant as they are considered central to the success of the USA300 MRSA clone, although naturally occurring deleted strains are not infrequently isolated [13, 22, 69]. We also encountered *agr* mutant strains, as have others who have studied *S. aureus* colonization [19, 59, 70]. These mutants may be evolutionary dead ends if transmission to new hosts preferentially selects for ACME+, SCC*mec* + and agr + strains.

This study had a number of limitations. Due to sampling design we were constrained to sequencing one colony at each anatomic site/time point and could not estimate the extent of within-strain variation on a single host [71]. In this regard, recent studies have shown that rare variants within a population can be important in tracking transmission among hosts [72, 73]. We also did not track colonization of household contacts, and therefore we do not know what role re-transmission among individuals played in prolonging the apparent colonization of an individual.

We have used comparative genomics to sketch out some of the features of *S. aureus* asymptomatic, longitudinal colonization of humans. There are many unanswered questions related to the evolution of *S. aureus* during colonization, transmission and disease. We do not know how long an ISL persists on individuals nor how effective various classes of systemic antibiotics are in reducing colonization. We know that individual clones persist for years in households, but there may be expansion of a reservoir with frequent transfer of strains between individuals and fomites [26, 27]. We do not know which body site is the most common source for transmission between carriers or between carriers and fomites and where on the body there are more likely to be transitions between colonizing and infecting *S. aureus*. It would also be invaluable to compare the *S. aureus* strain to the background skin microbiome in order to start to investigate the source of selective pressure to lose or gain genetic elements such as SCC*mec* and the selective pressure leading to persistence or loss of *S. aureus* colonization. Future research that address these questions will support the development of interventions to prevent *S. aureus* infections in susceptible people.

## Conclusions

Using genome sequencing we were able to plausibly define USA300 *S. aureus* ISLs. We showed colonization of USA300 after an index USA300 MRSA infection of a patient for up to a year. Persistently colonizing strains could be found in all anatomic sites tested: nose, throat, and perirectal area. We showed that colonization by USA300, including MSSA and MRSA, was less prevalent when sampled more than a year after an initial infection. During the course of colonization there were changes in plasmid and prophage content and antibiotic resistance, including loss the SCC*mec* cassette to create MSSA strains. Using similar genome-based approaches but with more intensive sampling, it may be possible to define evolutionary constraints giving rise to bottlenecks emerging after exposure to antimicrobials and to determine the natural history of asymptomatic colonization of people by pathogenic *S. aureus* strain types as well as perhaps their risk of recurrent infection.

## Additional files

Additional file 1: Patient metadata. Breakdown of all metadata fields collected and summary of genetic results. Includes antibiotics administered to patient during the study period. (XLSX 36 kb)

Additional file2: Core and recombinant regions of USA300 TCH1516. Sheet1 = list of core blocks identified by parsnp. Sheet2 = list of recombinant blocks in the core region identified by ClonalFrame (XLSX 59 kb)

Additional file 3: Additional figures and tables. **Table S1.** agr frameshifts. **Figure S1.** Provenance of strains sequenced in the study. **Figure S2.** USA300 ISL strains isolated from each body site. **Figure S3.** SNP frequency by genome. **Figure S4.** within ISL estimates for pangenome. **Figure S5.** Plasmid distribution. **Figure S6.** Phage distribution. (PDF 1040 kb)

Additional file 4: Antibiotic resistance phenotypes. Table of phenotypic antibiotic results for the initial infection isolates. (CSV 8 kb)

## Abbreviations

ACME: arginine catabolic mobile element
ISL: intra-subject lineage
MLST: multilocus sequence type
MRCA: most recent common ancestor
MRSA: methicillin resistant *S. aureus*
MSSA: methicillin sensitive *S. aureus*
PVL: Panton-Valentine leukocidin
SSTI: skin and soft tissue infection

## References

[1] Tong SYC, Davis JS, Eichenberger E, Holland TL, Fowler VG (2015). *Staphylococcus aureus* infections: epidemiology, pathophysiology, clinical manifestations, and management. Clin Microbiol Rev.

[2] Senn L, Clerc O, Zanetti G, Basset P, Prod’hom G, Gordon NC (2016). The stealthy superbug: the role of asymptomatic enteric carriage in maintaining a long-term hospital outbreak of ST228 methicillin-resistant Staphylococcus aureus. MBio.

[3] Wertheim HFL, Melles DC, Vos MC, van Leeuwen W, van Belkum A, Verbrugh HA (2005). The role of nasal carriage in Staphylococcus aureus infections. Lancet Infect Dis.

[4] Chambers HF, Deleo FR (2009). Waves of resistance: Staphylococcus aureus in the antibiotic era. Nat Rev Microbiol.

[5] Monecke S, Coombs G, Shore AC, Coleman DC, Akpaka P, Borg M (2011). A field guide to pandemic, epidemic and sporadic clones of methicillin-resistant Staphylococcus aureus. PLoS One.

[6] Liu J, Chen D, Peters BM, Li L, Li B, Xu Z (2016). Staphylococcal chromosomal cassettes mec (SCCmec): a mobile genetic element in methicillin-resistant Staphylococcus aureus. Microb Pathog.

[7] Jevons MP. “Celbenin” - resistant staphylococci. Br Med J BMJ Group; 1961 [cited 2017 2];1:124. Available from: https://www.ncbi.nlm.nih.gov/pmc/articles/PMC1952888/

[8] Harkins CP, Pichon B, Doumith M, Parkhill J, Westh H, Tomasz A (2017). Methicillin-resistant Staphylococcus aureus emerged long before the introduction of methicillin into clinical practice. Genome Biol.

[9] Fowler VG, Nelson CL, McIntyre LM, Kreiswirth BN, Monk A, Archer GL (2007). Potential associations between hematogenous complications and bacterial genotype in Staphylococcus aureus infection. J infect dis.

[10] Sharma-Kuinkel Batu K., Mongodin Emmanuel F., Myers Jason R., Vore Kelly L., Canfield Greg S., Fraser Claire M., Rude Thomas H., Fowler Vance G., Gill Steven R. (2015). Potential Influence ofStaphylococcus aureusClonal Complex 30 Genotype and Transcriptome on Hematogenous Infections. Open Forum Infectious Diseases.

[11] Li M, Diep BA, Villaruz AE, Braughton KR, Jiang X, DeLeo FR (2009). Evolution of virulence in epidemic community-associated methicillin-resistant Staphylococcus aureus. Proc Natl Acad Sci.

[12] Talan DA, Krishnadasan A, Gorwitz RJ, Fosheim GE, Limbago B, Albrecht V (2011). Comparison of Staphylococcus aureus from skin and soft-tissue infections in US emergency department patients, 2004 and 2008. Clin Infect Dis.

[13] Glaser P, Martins-Simões P, Villain A, Barbier M, Tristan A, Bouchier C (2016). Demography and intercontinental spread of the USA300 community-acquired methicillin-resistant Staphylococcus aureus lineage. MBio.

[14] Strauß L, Stegger M, Akpaka PE, Alabi A, Breurec S, Coombs G (2017). Origin, evolution, and global transmission of community-acquired Staphylococcus aureus ST8. Proc Natl Acad Sci U S A.

[15] David MZ, Daum RS (2010). Community-associated methicillin-resistant Staphylococcus aureus: epidemiology and clinical consequences of an emerging epidemic. Clin Microbiol Rev.

[16] Bowers JR, Driebe EM, Albrecht V, McDougal LK, Granade M, Roe CC, et al. Improved subtyping of Staphylococcus aureus clonal complex 8 strains based on whole-genome phylogenetic analysis. mSphere. 2018;3(3). pii: e00464-e00417. Available from: 10.1128/mSphere.00464-17 10.1128/mSphere.00464-17 PMC5932376 29720527

[17] Diep Binh An, Stone Gregory G., Basuino Li, Graber Christopher J., Miller Alita, Etages Shelley‐Ann des, Jones Alison, Palazzolo‐Ballance Amy M., Perdreau‐Remington Françoise, Sensabaugh George F., DeLeo Frank R., Chambers Henry F. (2008). The Arginine Catabolic Mobile Element and Staphylococcal Chromosomal CassettemecLinkage: Convergence of Virulence and Resistance in the USA300 Clone of Methicillin‐ResistantStaphylococcus aureus. The Journal of Infectious Diseases.

[18] Millar EV, Chen W-J, Schlett CD, Cui T, Crawford KB, Lanier JB (2015). Frequent use of chlorhexidine-based body wash associated with a reduction in methicillin-resistant Staphylococcus aureus nasal colonization among military trainees. Antimicrob Agents Chemother.

[19] Azarian T, Daum RS, Petty LA, Steinbeck JL, Yin Z, Nolan D (2016). Intrahost evolution of methicillin-resistant Staphylococcus aureus USA300 among individuals with reoccurring skin and soft-tissue infections. J Infect Dis [Internet].

[20] Millar EV, Rice GK, Elassal EM, Schlett CD, Bennett JW, Redden CL (2017). Genomic characterization of USA300 MRSA to evaluate Intraclass transmission and recurrence of SSTI among high risk military trainees. Clin Infect Dis.

[21] Planet PJ, Diaz L, Kolokotronis S-O, Narechania A, Reyes J, Xing G (2015). Parallel epidemics of community-associated methicillin-resistant Staphylococcus aureus USA300 infection in north and South America. J Infect Dis.

[22] Jamrozy DM, Harris SR, Mohamed N, Peacock SJ, Tan CY, Parkhill J, et al. Pan-genomic perspective on the evolution of the Staphylococcus aureus USA300 epidemic. Microb Genom Microbiology Society; 2016 [cited 2016 Apr 7];2:e000058. Available from: 10.1099/mgen.0.000058 10.1099/mgen.0.000058 PMC5320670 28348852

[23] Von Dach E, Diene SM, Fankhauser C, Schrenzel J, Harbarth S, François P (2016). Comparative genomics of community-associated methicillin-resistant Staphylococcus aureus shows the emergence of clone ST8-USA300 in Geneva, Switzerland. J Infect Dis.

[24] Price JR, Cole K, Bexley A, Kostiou V, Eyre DW, Golubchik T, et al. Transmission of Staphylococcus aureus between healthcare workers, the environment and patients in an intensive care unit: a whole-genome sequencing based longitudinal cohort study. Lancet Infect Dis Elsevier; 2016;17:207-14. [cited 2016 Nov 16]; Available from: http://discovery.ucl.ac.uk/1519622/10.1016/S1473-3099(16)30413-3 PMC5266793 27863959

[25] Sabat AJ, Hermelijn SM, Akkerboom V, Juliana A, Degener JE, Grundmann H (2017). Complete-genome sequencing elucidates outbreak dynamics of CA-MRSA USA300 (ST8-spa t008) in an academic hospital of Paramaribo, Republic of Suriname. Sci Rep.

[26] Alam MT, Read TD, Petit RA, Boyle-Vavra S, Miller LG, Eells SJ (2015). Transmission and microevolution of USA300 MRSA in U.S. households: evidence from whole-genome sequencing. MBio American Society for Microbiology.

[27] Uhlemann A.-C., Dordel J., Knox J. R., Raven K. E., Parkhill J., Holden M. T. G., Peacock S. J., Lowy F. D. (2014). Molecular tracing of the emergence, diversification, and transmission of S. aureus sequence type 8 in a New York community. Proceedings of the National Academy of Sciences.

[28] Toleman Michelle S., Reuter Sandra, Coll Francesc, Harrison Ewan M., Blane Beth, Brown Nicholas M., Török M. Estée, Parkhill Julian, Peacock Sharon J. (2016). Systematic Surveillance Detects Multiple Silent Introductions and Household Transmission of Methicillin-ResistantStaphylococcus aureusUSA300 in the East of England. Journal of Infectious Diseases.

[29] Harmsen D., Claus H., Witte W., Rothganger J., Claus H., Turnwald D., Vogel U. (2003). Typing of Methicillin-Resistant Staphylococcus aureus in a University Hospital Setting by Using Novel Software for spa Repeat Determination and Database Management. Journal of Clinical Microbiology.

[30] Horan TC, Andrus M, Dudeck MA (2008). CDC/NHSN surveillance definition of health care-associated infection and criteria for specific types of infections in the acute care setting. Am J Infect Control.

[31] Bankevich A, Nurk S, Antipov D, Gurevich AA, Dvorkin M, Kulikov AS (2012). SPAdes: a new genome assembly algorithm and its applications to single-cell sequencing. J Comput Biol.

[32] Seemann T (2014). Prokka: rapid prokaryotic genome annotation. Bioinformatics.

[33] Gordon NC, Price JR, Cole K, Everitt R, Morgan M, Finney J (2014). Prediction of Staphylococcus aureus antimicrobial resistance by whole-genome sequencing. J Clin Microbiol.

[34] Cingolani P, Platts A, Wang LL, Coon M, Nguyen T, Wang L (2012). A program for annotating and predicting the effects of single nucleotide polymorphisms, SnpEff: SNPs in the genome of *Drosophila melanogaster* strain w1118; iso-2; iso-3. Fly.

[35] Highlander SK, Hultén KG, Qin X, Jiang H, Yerrapragada S, Mason EO (2007). Subtle genetic changes enhance virulence of methicillin resistant and sensitive Staphylococcus aureus. BMC Microbiol.

[36] Pages H, Gentleman R, Aboyoun P, DebRoy S. Biostrings: string objects representing biological sequences, and matching algorithms, 2008. R package version. 2:160.

[37] Baba Tadashi, Takeuchi Fumihiko, Kuroda Makoto, Yuzawa Harumi, Aoki Ken-ichi, Oguchi Akio, Nagai Yoshimi, Iwama Natsuko, Asano Kazuyuki, Naimi Timothy, Kuroda Hiroko, Cui Longzhu, Yamamoto Kenji, Hiramatsu Keiichi (2002). Genome and virulence determinants of high virulence community-acquired MRSA. The Lancet.

[38] Camacho C, Coulouris G, Avagyan V, Ma N, Papadopoulos J, Bealer K (2009). BLAST+: architecture and applications. BMC Bioinformatics.

[39] Mi H, Poudel S, Muruganujan A, Casagrande JT, Thomas PD (2016). PANTHER version 10: expanded protein families and functions, and analysis tools. Nucleic Acids Res.

[40] Treangen TJ, Ondov BD, Koren S, Phillippy AM (2014). The harvest suite for rapid core-genome alignment and visualization of thousands of intraspecific microbial genomes. Genome Biol.

[41] Didelot X, Wilson DJ (2015). ClonalFrameML: efficient inference of recombination in whole bacterial genomes. PLoS Comput Biol.

[42] Liu K, Linder CR, Warnow T (2011). RAxML and FastTree: comparing two methods for large-scale maximum likelihood phylogeny estimation. PLoS One.

[43] Dress AWM, Flamm C, Fritzsch G, Grünewald S, Kruspe M, Prohaska SJ (2008). Noisy: identification of problematic columns in multiple sequence alignments. Algorithms Mol Biol.

[44] Pupko Tal, Pe Itsik, Shamir Ron, Graur Dan (2000). A Fast Algorithm for Joint Reconstruction of Ancestral Amino Acid Sequences. Molecular Biology and Evolution.

[45] Paradis E, Claude J, Strimmer K (2004). APE: analyses of Phylogenetics and evolution in R language. Bioinformatics.

[46] Goerke C, Pantucek R, Holtfreter S, Schulte B, Zink M, Grumann D (2009). Diversity of prophages in dominant Staphylococcus aureus clonal lineages. J Bacteriol.

[47] Lozano C, Garcia-Migura L, Aspiroz C, Zarazaga M, Torres C, Aarestrup FM (2012). Expansion of a plasmid classification system for gram-positive bacteria and determination of the diversity of plasmids in Staphylococcus aureus strains of human, animal, and food origins. Appl Environ Microbiol.

[48] David MZ, Acree ME, Sieth JJ, Boxrud DJ, Dobbins G, Lynfield R, et al. Pediatric S. aureus isolate genotypes and infections from the Dawn of the CA-MRSA epidemic era in Chicago, 1995-1997. J Clin Microbiol. 2015;53:2486-91. Available from: https://jcm.asm.org/content/53/8/2486. 10.1128/JCM.00096-15 PMC4508449 26019202

[49] Arredondo-Alonso S, van Schaik W, Willems RJ, Schurch AC. On the (im)possibility to reconstruct plasmids from whole genome short-read sequencing data. bioRxiv. 2016 [cited 2016 Nov 14]. p. 086744. Available from: http://biorxiv.org/content/early/2016/11/14/086744 10.1099/mgen.0.000128 PMC5695206 29177087

[50] Kuroda Makoto, Ohta Toshiko, Uchiyama Ikuo, Baba Tadashi, Yuzawa Harumi, Kobayashi Ichizo, Cui Longzhu, Oguchi Akio, Aoki Ken-ichi, Nagai Yoshimi, Lian JianQi, Ito Teruyo, Kanamori Mutsumi, Matsumaru Hiroyuki, Maruyama Atsushi, Murakami Hiroyuki, Hosoyama Akira, Mizutani-Ui Yoko, Takahashi Noriko K, Sawano Toshihiko, Inoue Ryu-ichi, Kaito Chikara, Sekimizu Kazuhisa, Hirakawa Hideki, Kuhara Satoru, Goto Susumu, Yabuzaki Junko, Kanehisa Minoru, Yamashita Atsushi, Oshima Kenshiro, Furuya Keiko, Yoshino Chie, Shiba Tadayoshi, Hattori Masahira, Ogasawara Naotake, Hayashi Hideo, Hiramatsu Keiichi (2001). Whole genome sequencing of meticillin-resistant Staphylococcus aureus. The Lancet.

[51] Diep BA, Gill SR, Chang RF, Phan TH, Chen JH, Davidson MG (2006). Complete genome sequence of USA300, an epidemic clone of community-acquired meticillin-resistant Staphylococcus aureus. Lancet.

[52] Jensen LB, Garcia-Migura L, Valenzuela AJS, Løhr M, Hasman H, Aarestrup FM (2010). A classification system for plasmids from enterococci and other gram-positive bacteria. J Microbiol Methods [Internet].

[53] Keggins K. M., Lovett P. S., Duvall E. J. (1978). Molecular cloning of genetically active fragments of Bacillus DNA in Bacillus subtilis and properties of the vector plasmid pUB110. Proceedings of the National Academy of Sciences.

[54] Ramsay JP, Kwong SM, Murphy RJT, Yui Eto K, Price KJ, Nguyen QT (2016). An updated view of plasmid conjugation and mobilization in staphylococcus. Mob Genet Elements.

[55] Khan Saleem A., Novick Richard P. (1983). Complete nucleotide sequence of pT181, a tetracycline-resistance plasmid from Staphylococcus aureus. Plasmid.

[56] Rocha EPC, Smith JM, Hurst LD, Holden MTG, Cooper JE, Smith NH (2006). Comparisons of dN/dS are time dependent for closely related bacterial genomes. J Theor Biol.

[57] Planet PJ, Larussa SJ, Dana A, Smith H, Xu A, Ryan C (2013). Emergence of the epidemic methicillin-resistant Staphylococcus aureus strain USA300 coincides with horizontal transfer of the arginine catabolic Mobile element and speG-mediated adaptations for survival on skin. MBio.

[58] Otto M (2013). Community-associated MRSA: what makes them special?. Int J Med Microbiol.

[59] Shopsin B, Drlica-Wagner A, Mathema B, Adhikari RP, Kreiswirth BN, Novick RP (2008). Prevalence of agr dysfunction among colonizing Staphylococcus aureus strains. J Infect Dis.

[60] Traber KE, Lee E, Benson S, Corrigan R, Cantera M, Shopsin B (2008). Agr function in clinical Staphylococcus aureus isolates. Microbiology.

[61] Young BC, Golubchik T, Batty EM, Fung R, Larner-Svensson H, Votintseva AA (2012). Evolutionary dynamics of Staphylococcus aureus during progression from carriage to disease. Proc Natl Acad Sci.

[62] Oh J, Byrd AL, Park M, Kong HH, Segre JA. Temporal stability of the human skin microbiome. Cell.;2016 [cited 2016 May 5];165:854–866. Available from: http://www.cell.com/cell/fulltext/S0092-8674(16)30399-3 10.1016/j.cell.2016.04.008 PMC4860256 27153496

[63] Harris SR, Feil EJ, Holden MTG, Quail MA, Nickerson EK, Chantratita N (2010). Evolution of MRSA during hospital transmission and intercontinental spread. Science.

[64] Holden MTG, Hsu LY, Kurt K, Weinert LA, Mather AE, Harris SR (2013). A genomic portrait of the emergence, evolution, and global spread of a methicillin-resistant Staphylococcus aureus pandemic. Genome Res.

[65] McAdam PR, Templeton KE, Edwards GF, Holden MTG, Feil EJ, Aanensen DM (2012). Molecular tracing of the emergence, adaptation, and transmission of hospital-associated methicillin-resistant Staphylococcus aureus. Proc Natl Acad Sci.

[66] Spoor LE, Mcadam PR, Weinert LA, Rambaut A, Hasman H, Aarestrup FM (2013). Livestock origin for a human pandemic clone of community-associated methicillin-resistant Staphylococcus aureus. MBio.

[67] Kluytmans J, van Belkum A, Verbrugh H (1997). Nasal carriage of Staphylococcus aureus: epidemiology, underlying mechanisms, and associated risks. Clinical Microbiology Reviews.

[68] Williamson DA, Ritchie S, Keren B, Harrington M, Thomas MG, Upton A (2016). Persistence, discordance and diversity of Staphylococcus aureus nasal and oropharyngeal colonization in school-aged children. Pediatr Infect Dis J.

[69] Ledda A, Price JR, Cole K, Llewelyn MJ, Kearns AM, Crook DW (2017). Re-emergence of methicillin susceptibility in a resistant lineage of Staphylococcus aureus. J Antimicrob Chemother.

[70] Smyth DS, Kafer JM, Wasserman GA, Velickovic L, Mathema B, Holzman RS (2012). Nasal carriage as a source of agr-defective Staphylococcus aureus bacteremia. J Infect Dis.

[71] Golubchik Tanya, Batty Elizabeth M., Miller Ruth R., Farr Helen, Young Bernadette C., Larner-Svensson Hanna, Fung Rowena, Godwin Heather, Knox Kyle, Votintseva Antonina, Everitt Richard G., Street Teresa, Cule Madeleine, Ip Camilla L. C., Didelot Xavier, Peto Timothy E. A., Harding Rosalind M., Wilson Daniel J., Crook Derrick W., Bowden Rory (2013). Within-Host Evolution of Staphylococcus aureus during Asymptomatic Carriage. PLoS ONE.

[72] Worby CJ, Lipsitch M, Hanage WP. Shared genomic variants: identification of transmission routes using pathogen deep sequence data. bioRxiv. 2015 [cited 2015 Nov 30]. p. 032458. Available from: http://biorxiv.org/content/early/2015/11/20/032458 10.1093/aje/kwx182 PMC5860558 29149252

[73] Worby CJ, Lipsitch M, Hanage WP (2014). Within-host bacterial diversity hinders accurate reconstruction of transmission networks from genomic distance data. PLoS Comput Biol.

[74] Letunic I, Bork P (2016). Interactive tree of life (iTOL) v3: an online tool for the display and annotation of phylogenetic and other trees. Nucleic Acids Res.

