# Supplementary material for: USA300 Staphylococcus aureus persists on multiple body sites following an infection
Source: BMC Microbiol. 2018 Dec 5;18:206. doi: 10.1186/s12866-018-1336-z (PMC6282268; doi:10.1186/s12866-018-1336-z)

Supplementary Table 1 *agr* frameshifts

Gene	TCH1516 locus_tag	Mutation	Sample_IDs	Patient/ samples
agrC	USA300HOU_RS10970	2149206 GAA>GA	26	Patient 26: III
agrC	USA300HOU_RS10970	2149482 AGG>AG	76	Patient 26: throat1
agrA	USA300HOU_RS10975	2150087 ATG>AG	41,87,88	Patient 11: peri1, nose2, throat2

Supplementary Figure 1. Provenance of strains sequenced in the study.

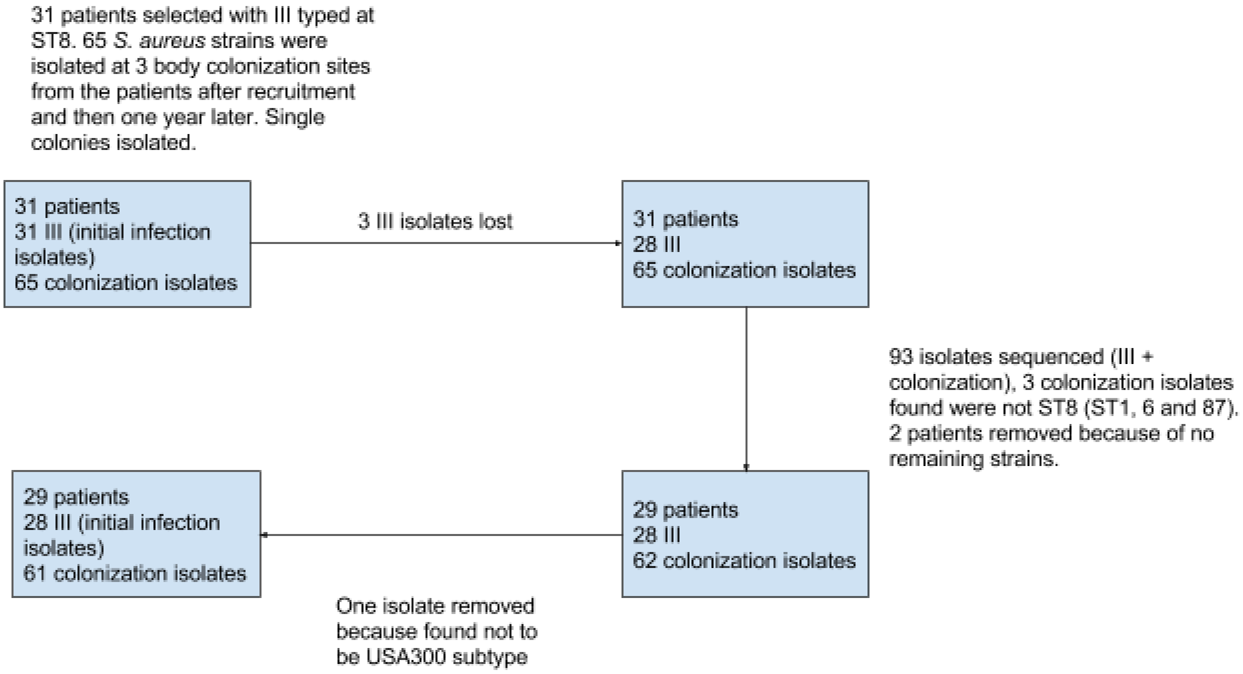

Supplementary Figure 2. USA300 ISL strains isolated from each body site

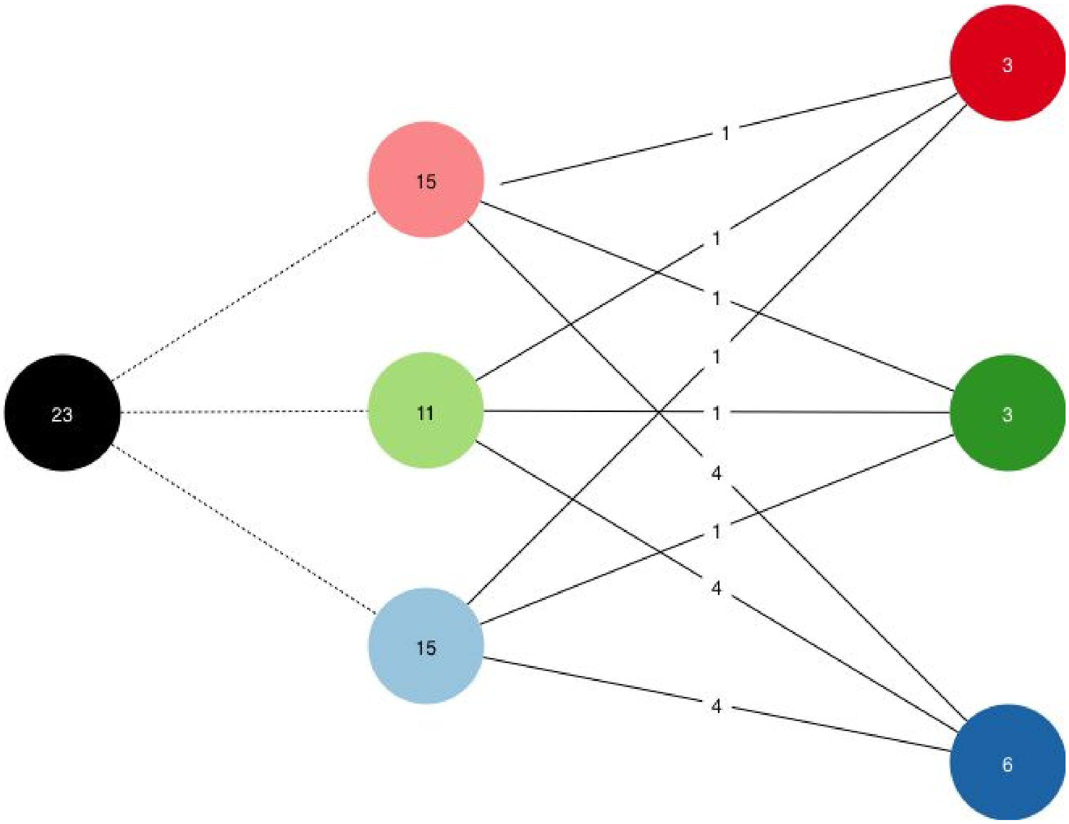

This figure uses the same color scheme as Figure 1 and S5, S6. Blue = nose, Green = Throat, Red = perirectal, black = III. Light colors are time 1, darker are time 2. Number in circle are the numbers of ISLs with each type. Connectors (not dashed) are the number of times an ISL contained both strain types.

Supplementary Figure 3. SNP frequency by genome

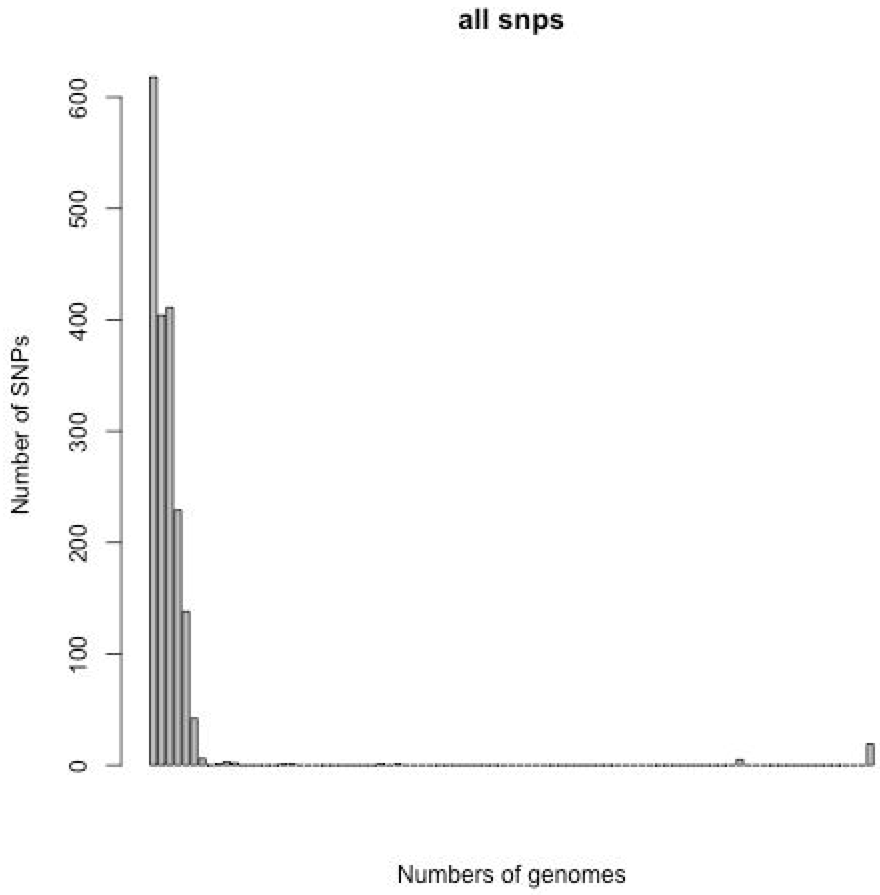

Supplementary Figure 4. Within ISL estimates for Pangenome

Using on FindMyFriends software (https://github.com/thomasp85/FindMyFriends). we estimated the total pangenome size to be 4,361 gene groups with a core of about 2,000 (Fig S2A). The total pangenome size is almost certainly inflated because of overcalling on the assembled from shotgun Illumina sequencing. Fig S2B. Strains with the same ISL were more similar to each other in terms of gene content than those in different patients/ISLs (median of 25 within-ISL compare to 120 between - figure below). Due to variability in plasmids and prophage regions we found as 500+ gene content changes between strains from the same recently infecting lineage.

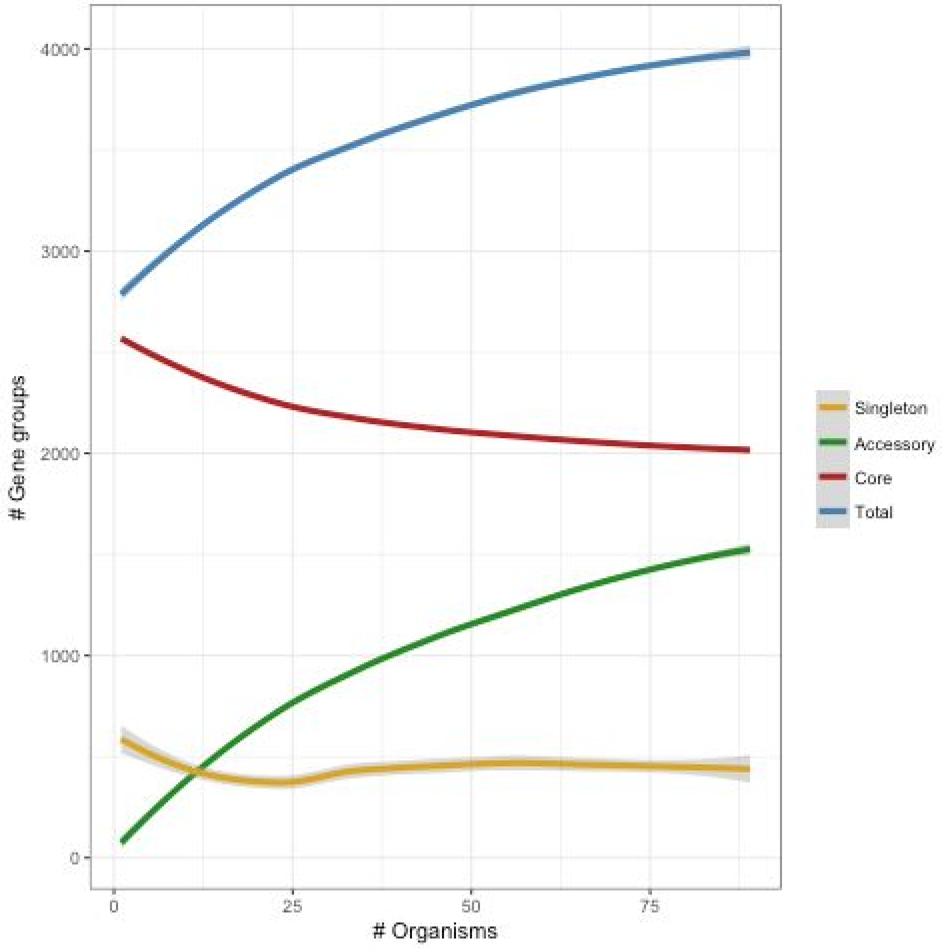

S4A

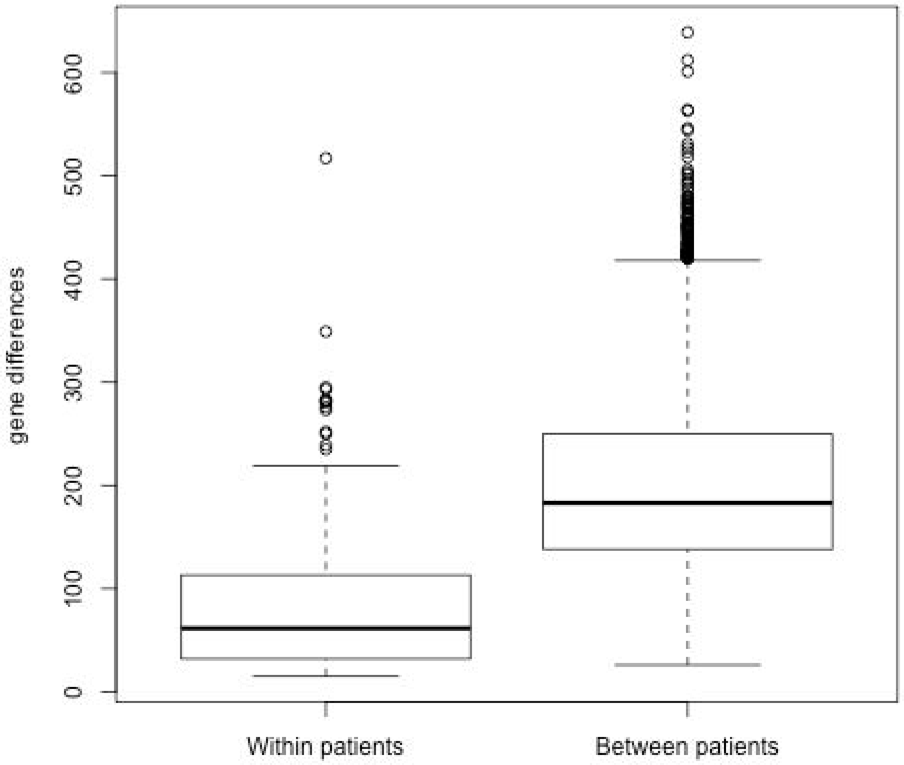

600
500
400
300
200
100
0
gene differences
Within patients
Between patients

S4B

Supplementary Figure 5. Plasmid distribution

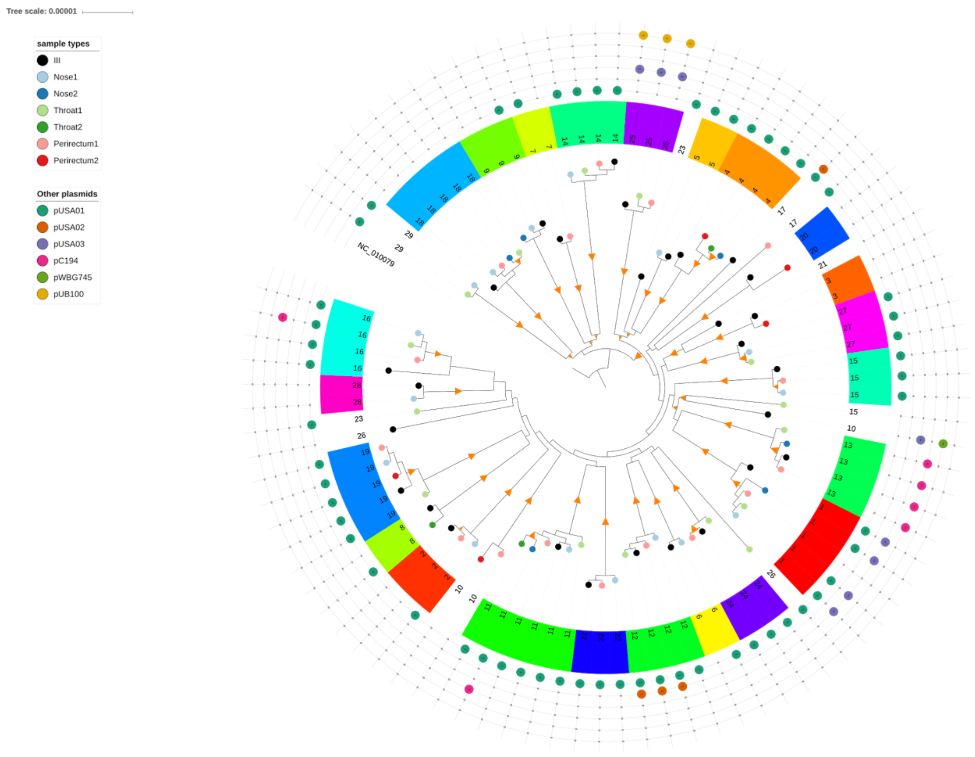

Supplementary Figure 6. Prophage distribution

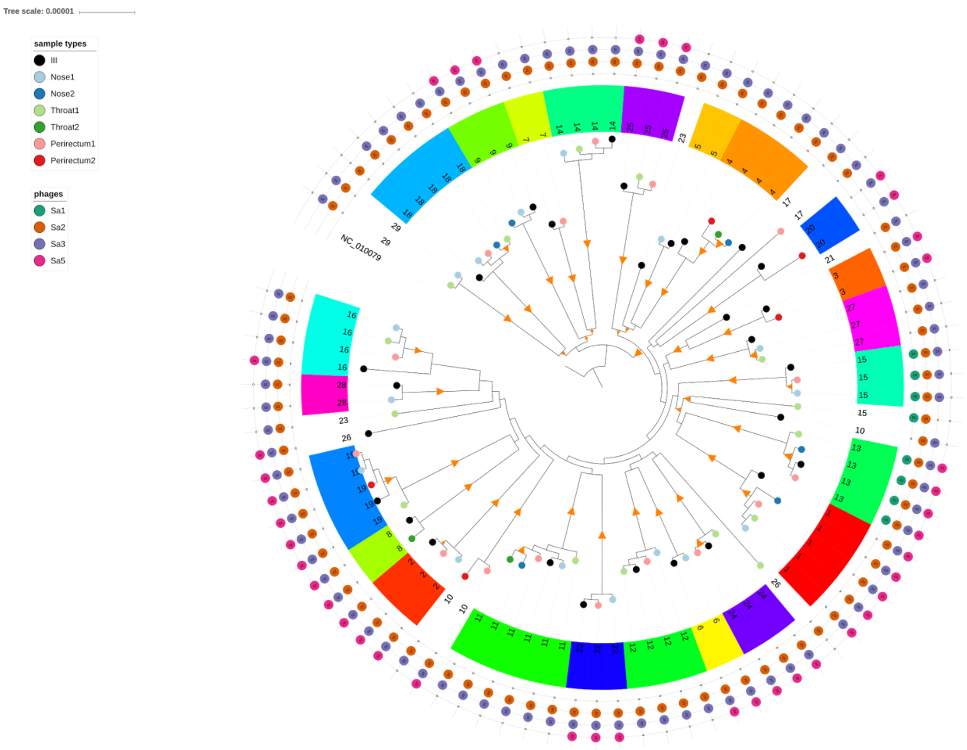

Supplement: Supplementary file 3 — Additional figures and tables. Table S1. agr frameshifts. Figure S1. Provenance of strains sequenced in the study. Figure S2. USA300 ISL strains isolated from each body site. Figure S3. SNP frequency by genome. Figure S4. within ISL estimates for pangenome. Figure S5. Plasmid distribution. Figure S6. Phage distribution. (PDF 1040 kb) [file 12866_2018_1336_MOESM3_ESM.pdf]
